# High expression of P-selectin induces neutrophil extracellular traps via the PSGL-1/Syk/Ca^2+^/PAD4 pathway to exacerbate acute pancreatitis

**DOI:** 10.3389/fimmu.2023.1265344

**Published:** 2023-09-28

**Authors:** Qi Xu, Ming Shi, Lu Ding, Yu Xia, Liang Luo, Xiaofang Lu, Xiaoying Zhang, David Y. B. Deng

**Affiliations:** ^1^ Department of Scientific Research Center, Seventh Affiliated Hospital, Sun Yat-Sen University, Shenzhen, China; ^2^ Department of Critical Care Medicine, Seventh Affiliated Hospital, Sun Yat-Sen University, Shenzhen, China; ^3^ Department of Pathology, Seventh Affiliated Hospital, Sun Yat-Sen University, Shenzhen, China; ^4^ Department of Health Management Center, Seventh Affiliated Hospital, Sun Yat-Sen University, Shenzhen, China

**Keywords:** acute pancreatitis, neutrophil extracellular traps, P-selectin, P-selectin glycoprotein ligand-1, peptidylarginine deiminase 4

## Abstract

**Background:**

Excessive neutrophil extracellular traps (NETs) is involved in the progression of acute pancreatitis (AP) but the mechanisms controlling NETs formation in AP are not fully understood. Therefore, our study sought to investigate the mechanism of the highly expressed P-selectin stimulating the formation of NETs in AP.

**Methods:**

NETs formation was detected by flow cytometry, immunofluorescence staining, and cf-DNA and MPO-DNA complexes were measured as biomarkers of NETs formation. Neutrophils treated with P-selectin and pharmacological inhibitors were examined by western blot, immunofluorescence staining and flow cytometry. Mouse model of AP was established by caerulein and the effect of inhibiting P-selectin by PSI-697 on the level of NETs and PAD4 in pancreatic tissue was observed. The severity of AP was evaluated by histopathological score and the detection of serum amylase and lipase.

**Results:**

Patients with AP had elevated levels of NETs and P-selectin compared with healthy volunteers. Stimulation of P-selectin up-regulated the expression of PSGL-1, increased the phosphorylation of Syk, mediated intracellular calcium signal and led to the activation and expression of PAD4, which modulated NETs formation in neutrophils. Pretreament with PSI-697 blunted NETs formation and PAD4 expression in the pancreatic tissue, and ameliorated the severity of AP in mice.

**Conclusion:**

Taken together, these results suggest that P-selectin induces NETs through PSGL-1 and its downstream Syk/Ca^2+^/PAD4 signaling pathway, and that targeting this pathway might be a promising strategy for the treatment of AP.

## 1 Introduction

Acute pancreatitis (AP) is an acute abdominal condition that is characterized by local and systemic inflammatory response and has a varying clinical course (1). The majority of patients present with mild acute pancreatitis (MAP) which is usually self-limiting and is characterized by pancreatic edema. Unfortunately, approximately 20% of AP patients develop severe acute pancreatitis (SAP), accompanied by pancreatic or peripancreatic tissue necrosis and even organ failure, which leads to a mortality rate of 20-40% (2). The treatment of patients suffering from AP is a great challenge and is largely limited to supportive therapies, which is partly related to the limited understanding of the pathophysiology that drives the progression of this disease.

Neutrophils appear to be the first responder cells that infiltrate to the site of injury and lead to progression towards SAP (3). A Study of AP patients with multiple organ dysfunction (MODS) and immunosuppression found increased neutrophil transmigration and aberrant signaling properties, suggesting that neutrophils contribute not only to the early phase of AP but also to the severe stage of multiple organ damages (4). Activated neutrophils also participate in AP by releasing NETs, which are large extracellular reticulate fibers composed of DNA, histones and granule proteins (5, 6). Studies have shown that NETs and their components can mediate pancreatic enzyme activation and exacerbate pancreatic inflammation and tissue damage in AP (7, 8). It has also been shown that the alkaline environment of pancreatic fluid can promote excessive NETs formation, which can form visible aggregates in the pancreatic ducts thereby blocking the ducts and aggravating pancreatic injury (9). Therefore, clarifying the stimuli and the signaling mechanisms controlling NETs formation in AP is helpful to provide a new basis for AP treatment.

Neutrophil infiltration into pancreatic tissue is a multistep process coordinated by specific adhesion molecules (10, 11) and evidence shows that inhibition of neutrophils or specific adhesion molecules may have a therapeutic effect on AP (12–14). P-selectin, a member of the adhesion molecules, is normally stored in granular structures of α-granules of platelets and Weibel-Palade bodies of endothelial cells and can be rapidly mobilized to the cell surface upon stimulation (15). It has been reported that serum P-selectin levels were significantly higher in AP patients on admission compared to healthy volunteers (16–18), and P-selectin levels were positively correlated with length of hospital stay (19). The conventional view is that P-selectin links inflammation and coagulation and plays a unique effect on the course of AP. Interestingly, there is a study that reported that P-selectin can promote NETs formation in neutrophils of mice (20). Based on the considerations above, we speculate that in addition to mediating inflammation and coagulation, high expression of P-selectin may contribute to AP deterioration by inducing the formation of excessive NETs, and the mechanism will be investigated.

Here, we chose AP mice and neutrophils isolated from human peripheral blood to study the receptor and signaling pathway that mediate the highly expressed P-selectin-induced NETs formation in AP. Our study revealed the role of PSGL-1 and its downstream Syk/Ca^2+^/PAD4 pathway in inducing NETs formation, providing a better understanding of the pathophysiological mechanisms of P-selectin in AP.

## 2 Methods

### 2.1 Patients

Patients who met at least two of the following three features were enrolled (1): Abdominal pain in compliance with AP (acute, sudden, persistent, severe upper abdominal pain, often radiating to the back) (2); Serum amylase and/or lipase activity at least three times higher than the upper limit of normal (3); characterized abdominal image manifestations of AP. Twelve patients with AP were included in AP group and healthy volunteers formed the control group. Peripheral blood samples were taken from AP patients within 24 h from the onset of pain. This study was approved by the Ethics Committee of the Seventh Affiliated Hospital of Sun Yat-Sen University. All persons gave their informed consent prior to their inclusion in the study.

### 2.2 Peripheral blood neutrophil isolation

Fresh peripheral blood was collected into EDTA tubes. The whole undiluted blood was then fractionated by density-gradient centrifugation using human peripheral blood neutrophil isolation kit (Solarbio, China) according to the manufacturer’s instruction. Neutrophil purity was established to be routinely >96%, as assessed by Wright-Giemsa staining. Purified neutrophils were resuspended in RPMI 1640 medium (Sigma, Sweden) supplemented with 2% (vol/vol) Fetal bovine serum (FBS) (BI, Israel). All operations were performed under sterile conditions.

### 2.3 Measurements of ex vivo NETs formation in AP patients

#### 2.3.1 Flow cytometry

Isolated neutrophils were fixed in 4% (vol/vol) paraformaldehyde (PFA) (biosharp, China), blocked with 3% (wt/vol) bovine serum albumin (BSA) (Solarbio, China) for 30min at 37°C and then incubated with the primary antibody Histone H3 (citrulline R2+R8+R17, ab5103, 1:300, abcam, USA), Alexa Fluor 647 conjugated secondary antibody (1:1000, Invitrogen, USA) at 1:300 dilution and FITC anti-myeloperoxidase antibody (ab11729, 1:10, Abcam) according to a previously described protocol (21). Flow cytometric analysis was operated using an Beckman CytoFLEX flow cytometer.

#### 2.3.2 Immunofluorescence staining

Purified neutrophils were seeded onto coverslips coated with 0.001% poly-L-lysine (Beyotime, China). Neutrophils were fixed in 4% (vol/vol) PFA for 30 min at room temperature, washed with PBS and permeabilized with 0.1% Triton X-100 for 10 min at 4°C. After blocking with 3% (wt/vol) BSA for 90 min at 37°C, neutrophils were incubated overnight at 4°C with primary antibody Histone H3 (citrulline R2+R8+R17, ab5103, 1:1000, Abcam, USA) and Myeloperoxidase (ab25989, 1:1000, Abcam, USA). Neutrophils were subsequently incubated with Alexa Fluor 568/647 conjugated secondary antibodies (1:1000, Invitrogen, USA) for 1 h at room temperature followed by DAPI solution for 10 min. NETs formation was visualized using a confocal microscope (Carl Zeiss, Jena, Germany).

### 2.4 Enzyme-linked immunosorbent assay

Levels of P-selectin in serums were detected by ELISA. The ELISA kit was purchased from FineTest (Wuhan, China). Specimens were diluted to 100 μL (1: 20) and measured at an optical density (OD) of 450 nm.

### 2.5 NETs induction and quantification *in vitro*

Neutrophils were stimulated with P-selectin recombinant protein (Sinobiological, China). After 4 h of incubation, supernatants were collected. The level of cell-free DNA (cf-DNA) in the supernatants was quantified using Quant-iT™ PicoGreen^®^ dsDNA Assay Kit (Invitrogen, USA) according to the manufacturer’s instructions. In addition, the concentration of MPO-DNA complexes was measured. Briefly, samples were incubated in a 96-well flat-bottom plate (component of a human MPO ELISA kit, Finetest, China) precoated with anti-MPO antibody. MPO as a constituent of NETs in mouse serum was captured, and the concentration of DNA bound to MPO was measured using the Quant-iT™ PicoGreen^®^ dsDNA Assay Kit (Invitrogen, USA) (22). Immunofluorescence staining mentioned above was used to observe the formation of NETs in neutrophils.

### 2.6 Analysis of Ca^2+^ in neutrophils

Fluo-4 AM (meilunbio, China) working solution was prepared by diluting Fluo-4 AM solution with HBSS (Gibco, USA) to 5 µM. The working solution was added to the neutrophils, incubated at 37°C for 40 min and then neutrophils were washed 3 times with 10 mM HEPES. After resting in 37°C for 20 min, neutrophils were treated in groups and analyzed by flow cytometry or visualized under a fluorescence microscope (Leica, Germany).

### 2.7 Protein extraction and western blotting

The total protein was extracted in ice-cold RIPA lysis buffer (Solarbio, China) containing PMSF (Solarbio, China) and Phosphatase Inhibitor Cocktail (Epizyme, China) and then sonicated and centrifuged (12,000 ×g for 15 min, 4°C) to collect supernatants. Protein concentration was detected with a BCA kit (Vazyme, China). Lysates of cells or pancreatic tissue were separated by electrophoresis at 10 or 12.5% SDS-polyacrylamide gel and transferred to Immobilon-P membrane (Millipore, USA) which was blocked with 5% BSA for 1 h at room temperature. Primary antibodies applied were as follows: PSGL-1 (NB100-78039, 1:500, NOVUS Biologicals, USA), p-Syk (AF8404, 1:2000, Affinity, USA), PAD4 (ab214810, 1:1000, Abcam, USA), Histone H3 (citrulline R2+R8+R17, ab5103, 1:1000, Abcam, USA), GAPDH (as internal control, 10494-1-AP, 1:5000, Proteintech, China) and β-actin (as internal control, 20536-1-AP, 1:5000, Proteintech, China). ECL detection system was used to visualized the protein bands on the membrane and the grayscale analysis of bands was calculated using ImageJ.

### 2.8 Detection of ROS generation

Dichloro-dihydrofluorescein diacetate probe (DCFH-DA) (Beyotime, China) was used to detect ROS in neutrophils. According to the manufacturer’s instructions, neutrophils were incubated in diluted DCFH-DA (1:5000 with serum-free 1640) at 37°C for 15 min, shielded from light. After three washes, a flow cytometry experiment was carried out to determine ROS generation.

### 2.9 Induction of AP models and drug administration

All animal experimental procedures were approved by the Ethics Committee of the Shenzhen TopBiotech Co. Ltd (NO. TOP-IACUC-2021-0129). Healthy adult male KM mice weighing between 20 and 25 g were purchased from the Shenzhen TopBiotech Co. Ltd (Shenzhen, China). All mice were housed in specific pathogen-free (SPF) facilities, fed standard rodent chow and water, and maintained at a controlled temperature (25 ± 2°C) under a light cycle (12 h light/12 h dark). AP model was induced by intraperitoneal injection of caerulein (50 μg/kg, MedChemExpress, USA) seven times at an interval of 1 h. Vehicle or PSI-697(30 mg/kg, intragastric administration, MedChemExpress, USA) was given 1 h before the first injection of caerulein. Animals were sacrificed 18 h after induction of AP and blood and pancreatic samples were collected. Blood were centrifuged at 4°C, 3000 rpm for 10 min to obtain serum. Serum amylase and lipase levels were detected by the use of a VITROS 5.1 FS automatic biochemistry analyzer (Johnson & Johnson).

### 2.10 Measurements of *in vivo* NETs formation

The levels of cf-DNA and MPO-DNA complexes were measured using MPO ELISA kit (Finetest, China) and dsDNA Assay Kit (Invitrogen, USA) as previously described. Mouse pancreas was freshly collected, fixed with 10% neutral buffered formalin and embedded in paraffin. Pancreatic tissues were sectioned into 3 μm. After antigen repair with citric acid-containing antigen repair buffer under high pressure, pancreatic tissue sections were blocked with 5% (wt/vol) BSA and incubated with the primary antibody Histone H3 (citrulline R2+R8+R17, ab5103, 1:1000, Abcam, USA) and Myeloperoxidase (GB12224, 1:1000, Servicebio, China) overnight at 4°C. After three washes, the sections were subsequently incubated with Alexa Fluor 568/647 conjugated secondary antibodies (1:1000, Invitrogen, USA) for 1 h at room temperature and counterstained with DAPI (1:2000, Sigma, Sweden).

### 2.11 Immunohistochemistry

After antigen repair, pancreatic tissue sections were incubated with 3%(vol/vol) hydrogen peroxide solution for 10 min to block endogenous peroxidase activity and then were blocked by goat serum for 30 min at room temperature. The sections were incubated with the primary antibody P-selectin (DF13294, 1:500, Affinity, USA) overnight at 4°C and then biotinylated secondary antibodies for 1 h at room temperature. Finally, sections were counterstained with hematoxylin.

### 2.12 Immunofluorescence staining

Antigen repair and blocking were performed on the Pancreatic tissue sections as previously described. Then the sections were incubated with the primary antibody PAD4 (ab214801, 1:500, Abcam, USA) and Ly6G (BE0075-1, 1:200, InVivoMAb, USA) overnight at 4°C. After three washes, the sections were subsequently incubated with Alexa Fluor 568/647 conjugated secondary antibodies (1:1000, Invitrogen, USA) for 1 h at room temperature and counterstained with DAPI (1:2000, Sigma, Sweden).

### 2.13 Analysis of mRNA expression

The expression level of PAD4 (forward primer: 5’-GACCCGAAAGCTCTATATGTCA-3’; reverse primer: 5’-TATTCCCGATGAGAATTCTGCC-3’) was measured using quantitative reverse transcription polymerase chain reaction (qRT-PCR) and GAPDH was used as internal control. RNA was extracted from mouse pancreatic tissue and reverse-transcribed according to the manufacturer’s protocol. qRT-PCR was performed with cDNA per sample per target gene using Taq Pro Universal SYBR qPCR Master Mix to measure the signal intensity. Relative fold changes in mRNA expression were quantified with the 2^−ΔΔCt^.

### 2.14 Histopathological severity evaluation

Pancreatic tissues were stained with hematoxylin and eosin (H&E). Histomorphological damage to the pancreas was assessed by the severity of edema, necrosis and inflammation (23) (**Supplementary Table 1**). All the histopathological evaluation was scored by two independent pathologists.

### 2.15 Statistical analysis

Data were presented as the mean ± the standard error of the mean (SEM). Statistical analysis was performed using Student’s two tailed t-test for comparisons of two groups or one-way ANOVA, Kruskal-Wallis test or Welch’s ANOVA for multiple groups. All statistical analyses were performed with GraphPad Prism 8 software (GraphPad, San Diego, CA, USA). *P*<0.05 was considered statistically significant, and n represents the number of animals in each group.

## 3 Results

### 3.1 Excessive NETs formation and hyperexpression of P-selectin in AP patients

**Figure 1:**
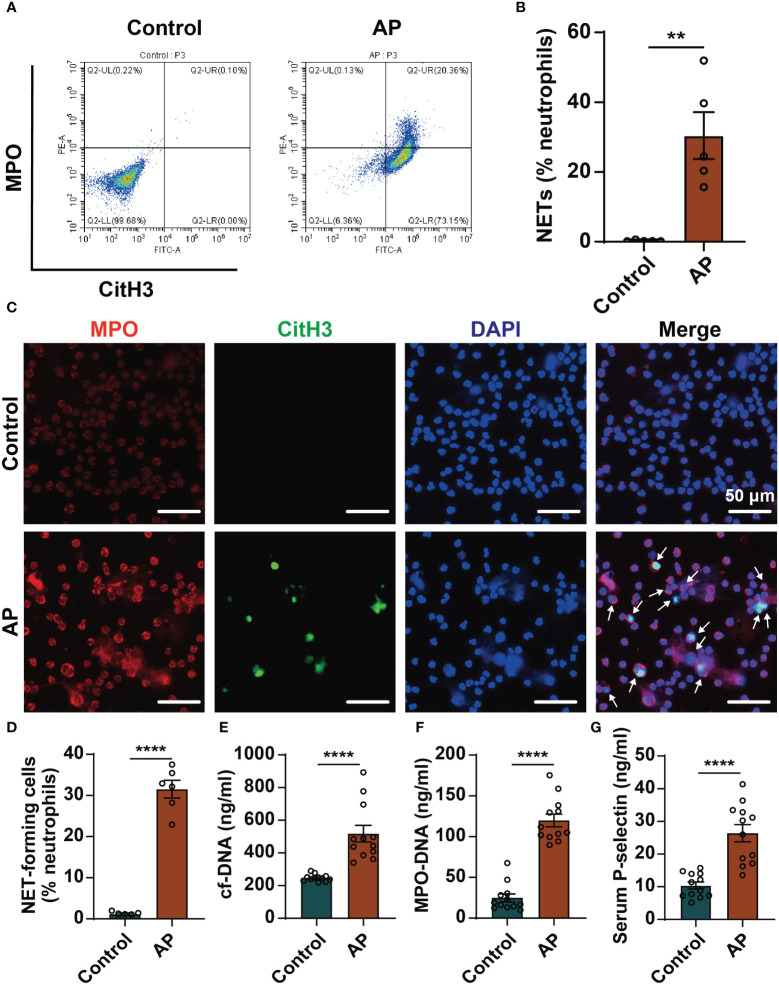
Elevated levels of NETs and P-selectin in AP patients. **(A)** Peripheral blood was collected from healthy individuals and AP patients. Neutrophils were isolated, and NETs formation was detected by flow cytometry. Representative flow data showing CitH3-positive and MPO-positive cells defined as NETs. **(B)** Quantification of circulating NETs in healthy controls and AP patients. (n=5, **P < 0.01) **(C)** Representative images of neutrophils stained with MPO (red), CitH3 (Green) and DAPI (blue). Scale bar: 50 μm. The white arrows indicated NET-forming cells. **(D)** The proportion of NET-forming cells in total neutrophils were quantified. (n=6, ****P < 0.0001) The serum levels of cf-DNA **(E)** and MPO-DNA complexes **(F)** were determined in serums of healthy individuals and AP patients. (n=12, ****P < 0.0001) **(G)** Serum levels of P-selectin in the control group and AP patients determined by ELISA. (n=12, ****P < 0.0001).

We detected the level of NETs formation in blood samples of AP patients by flow cytometry. The ratio of NETs in peripheral blood neutrophils was significantly higher in patients with AP compared with healthy individuals (**Figures 1A, B**). Likewise, immunofluorescence staining was performed on neutrophils isolated from blood samples. A large number of NETs structures appeared in AP group, which was almost invisible in control group (**Figures 1C, D**). It was also found that in the serums of AP patients the levels of cf-DNA and MPO-DNA complexes, two markers of NETs, were significantly higher than those of the control group (**Figures 1E, F**). Furthermore, the occurrence of AP markedly increased the levels of P-selectin in serums (**Figure 1G**). Consistently, high levels of P-selectin in pancreatic tissue and serums were also detected in mouse AP model (**Supplementary Figure 1**). These results support that the levels of NETs and P-selectin increased in AP. The relationship between the high expression of P-selectin and the excessive formation of NETs in AP would be further explored.

### 3.2 High level of P-selectin induces NETs formation in human neutrophils

**Figure 2:**
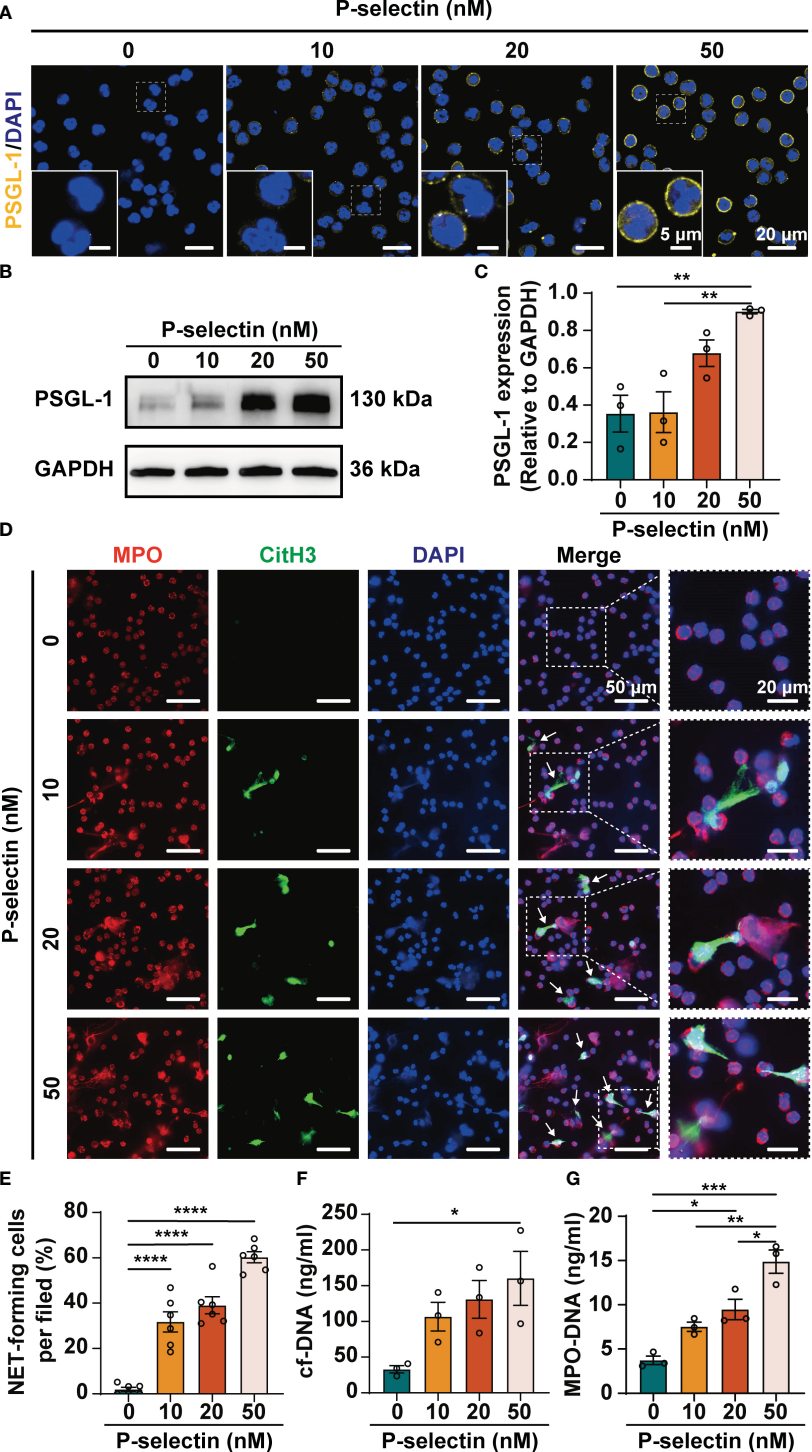
High level of P-selectin leads to increased PSGL-1 expression and NETs formation in human neutrophils. Human peripheral blood neutrophils were isolated and treated with P-selectin recombinant protein at concentrations of 10, 20 and 50 nM. **(A)** Representative images of neutrophil stained with PSGL-1 (yellow) and DAPI (blue). The inset box from each group is magnified. Scale bar: 5 μm and 20 μm, respectively. **(B)** Cell lysates were collected and subjected to Western blot analysis for PSGL-1. GAPDH was used as a loading control. Data from one representative experiment are shown. **(C)** Relative intensities of PSGL-1 against GAPDH. (n=3, **P < 0.01) **(D)** Representative images of neutrophils stained with MPO (red), CitH3 (Green) and DAPI (blue). The inset box from each group is magnified. Scale bar: 50 μm and 20 μm, respectively. The arrows indicate NETs. **(E)** NET-forming cells per field are quantified. (n=6, ****P < 0.0001) The neutrophil supernatants were assessed for cf-DNA **(F)** and MPO-DNA complexes **(G)**. (n=3, *P < 0.05, **P < 0.01, ***P < 0.001).

Based on the verification of hyperexpression of P-selectin and excessive NETs formation in AP, we further investigated how P-selectin affects NETs formation in human neutrophils. Neutrophils were isolated from peripheral blood of healthy volunteers and treated with P-selectin recombinant protein at different concentrations. Fluorescence images showed that PSGL-1 expression was up-regulated on the surface of neutrophils under the stimulation of P-selectin (**Figure 2A**). Western blot was also used to determine the expression of PSGL-1, and the results showed that the expression of this ligand increased to varying degrees upon stimulation with different concentrations of P-selectin (**Figures 2B, C**). Immunofluorescence analysis demonstrated that stimulation of P-selectin induced extracellular DNA traps formation in a dose-dependent manner, and high level of P-selectin recombinant protein (50 nM) stimulated approximately 60% of neutrophils to form NETs (**Figures 2D, E**). To objectively quantify the increased extracellular DNA visualized during *in vitro* NETs formation, cf-DNA (**Figure 2F**) and MPO-DNA complexes (**Figure 2G**) in the supernatants were measured. When the concentration of P-selectin reached 50 nM, the levels of these two substances were significantly higher than that of the control group. Hence, we used 50 nM as the stimulation concentration in subsequent *in vitro* experiments.

### 3.3 High level of P-selectin induces NETs formation through Syk/Ca*2+*/PAD4 signaling pathway

**Figure 3:**
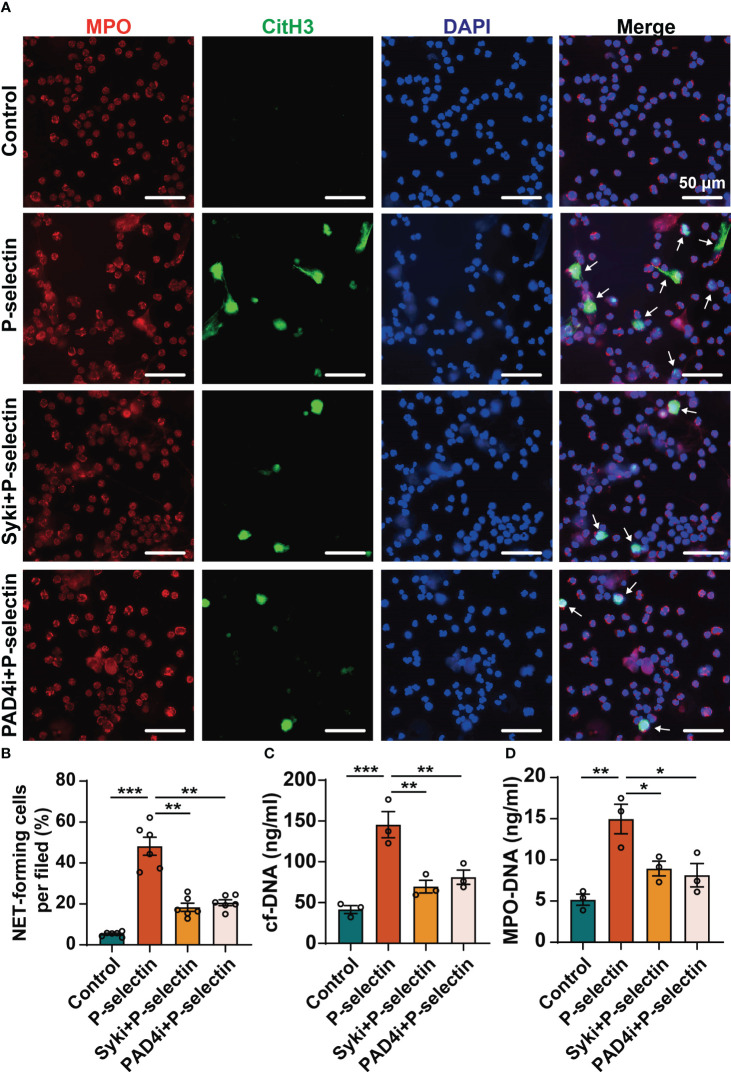
High level of P-selectin-induced NETs are regulated by Syk and PAD4. Neutrophils were pre-treated with Syk inhibitor (Syki, 4 μM of PRT-060318) or PAD4 inhibitor (PAD4i, 10 μM of GSK484) for 1 h before treated with P-selectin for 4 h. **(A)** Neutrophils were immune-stained with Abs to MPO, CitH3, and DAPI nuclear stain. Scale bar: 50 μm. The arrows indicate NETs. **(B)** NET-forming cells per field are quantified. (n=6, **P < 0.01, ***P < 0.001) The neutrophil supernatants were assessed for cf-DNA **(C)** and MPO-DNA complexes **(D)**. (n=3, *P < 0.05, **P < 0.01, ***P < 0.001).

**Figure 4:**
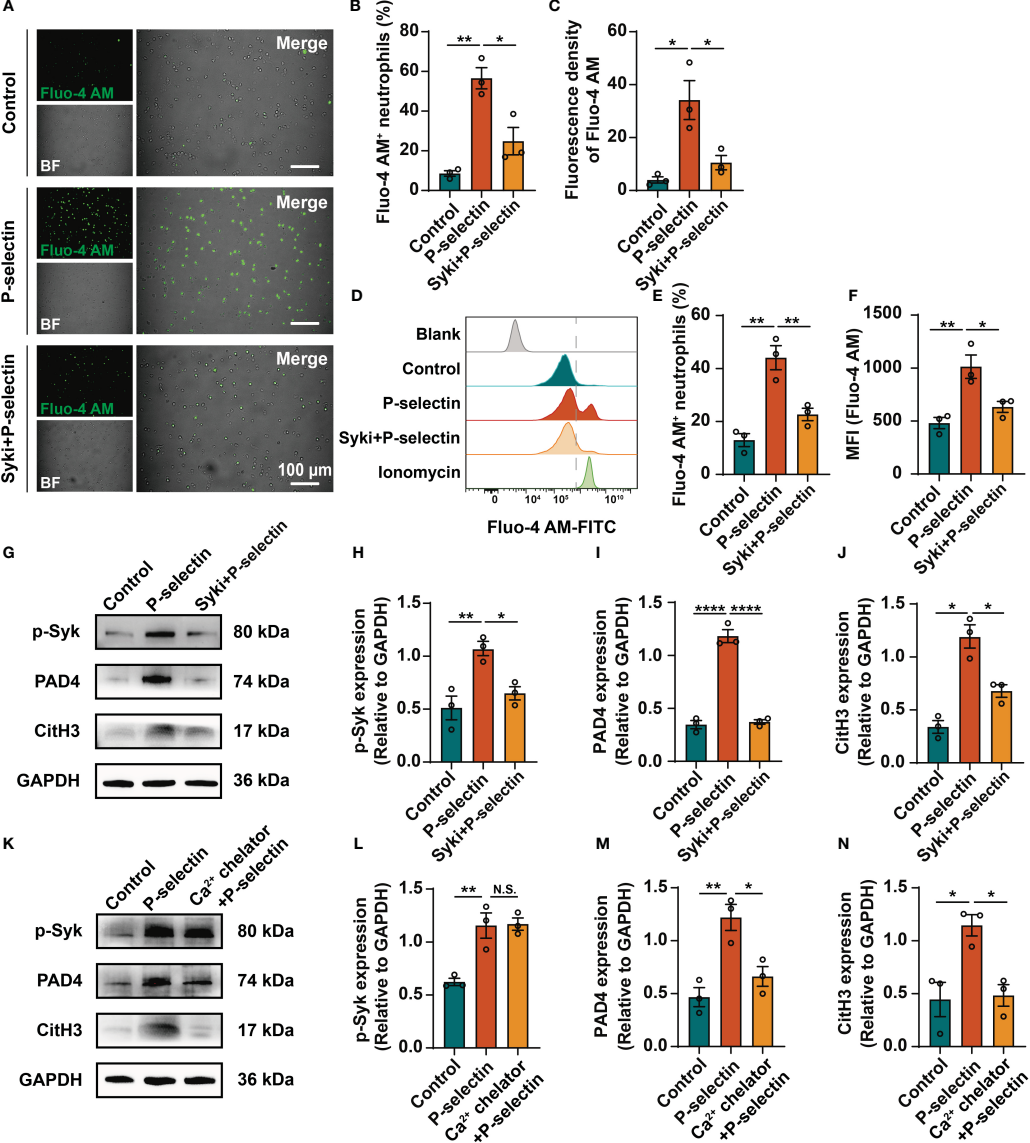
High level of P-selectin induces NETs formation through Syk/Ca^2+^/PAD4 signaling pathway. Neutrophils were pretreated with Syk inhibitor (Syki, 4 μM of PRT-060318) or Ca2+ chelator (3 μM of BAPTA-AM) for 1h and then were stimulated with P-selectin recombinant protein. Neutrophils were loaded with Ca^2+^ fluorescence probe Fluo-4 AM and then were analyzed by fluorescence microscopy and flow cytometry. **(A)** Representative images of neutrophils labeled by Fluo-4 AM under fluorescence microscope. Scale bar: 100 μm. **(B, C)** Quantitative analysis of the proportion of Fluo-4 AM^+^ neutrophils and fluorescence density of Fluo-4 AM under fluorescence microscope. (n=3, *P < 0.05, **P < 0.01) **(D)** Flow cytometry observation and quantification of Fluo-4 AM in neutrophils in different groups. Neutrophils treated with ionomycin were used as positive control. **(E, F)** Quantitative analysis of the Fluo-4 AM^+^ neutrophils ratio and the mean fluorescence intensity (MFI) detected by flow cytometry. (n=3, *P < 0.05, **P < 0.01) Neutrophils were pretreated with Syk inhibitor **(G)** or Ca^2+^ chelator **(K)** for 1h and then were stimulated with P-selectin recombinant protein. Cell lysates were collected and subject to Western blot analysis for p-Syk, PAD4 and CitH3. GAPDH was used as loading control. **(H–J, L–N)** Relative intensities are quantified by ImageJ. (n=3, N.S. represents no significant difference, *P < 0.05, **P < 0.01, ****P < 0.0001).

We used pharmacological inhibitors to inhibit the downstream key molecules of P-selectin and PSGL-1, and found that inhibiting Syk by PRT-060318 significantly reduced the formation of NETs. Administration of PRT-060318 significantly prevented NETs formation as demonstrated by decreased MPO/CitH3 colocalization (**Figures 3A, B**). Additionally, the results of dsDNA and ELISA assay of neutrophil supernatants demonstrated that PRT-060318 impaired extracellular release of cf-DNA and MPO-DNA complexes caused by stimulation of P-selectin (**Figures 3C, D**). In addition, inhibiting the key enzyme PAD4 which catalyzes the citrullinization of histone by GSK484 could also diminish the formation of NETs (**Figures 3A–D**). These results together indicated that Syk and PAD4 are involved in P-selectin-induced NETs. Since the activation of PAD4 requires the participation of calcium ions, we detected the intracellular Ca^2+^ and found that stimulation of P-selectin increased the calcium signal in neutrophils, while neutrophils pretreated with PRT-060318 before stimulation reduced the proportion of Ca^2+^-positive cells and the fluorescence intensity of Ca^2+^ probe (**Figures 4A–F**). Western blot also showed that neutrophils treated with P-selectin increased the phosphorylation of Syk and lifted the levels of PAD4 and CitH3 expression. Neutrophils treated with Syk inhibitor PRT-060318 before stimulation had lower expression of both PAD4 and CitH3 (**Figures 4G–J**), while neutrophils treated with Ca^2+^ chelator BAPTA-AM had lower levels of PAD4 and CitH3 but not that of phosphorylated-Syk (p-Syk) (**Figures 4K–N**). These results revealed that P-selectin might induces NETs formation through Syk/Ca^2+^/PAD4 pathway. Since ROS production is well-described to be involved in NETosis, we also studied the role of ROS in P-selectin-induced NETs in human neutrophils. Firstly, we used the ROS indicator DCFH-DA to detect the ROS level in neutrophils. The results showed that the stimulation of P-selectin did not lead to the increase of ROS level in human neutrophils (**Supplementary Figure 2A**). Secondly, we used Tempol as a ROS scavenger for neutrophils, and found that Tempol had little effect on the formation of NETs induced by P-selectin (**Supplementary Figure 2B**).These results suggest that ROS may not be involved in the signaling pathway of the formation of NETs in human neutrophils induced by P-selectin (**Supplementary Figure 2**)

### 3.4 Inhibition of P-selectin binding to PSGL-1 reduces the level of NETs and the expression of PAD4 in the pancreatic tissue of AP mice

**Figure 5:**
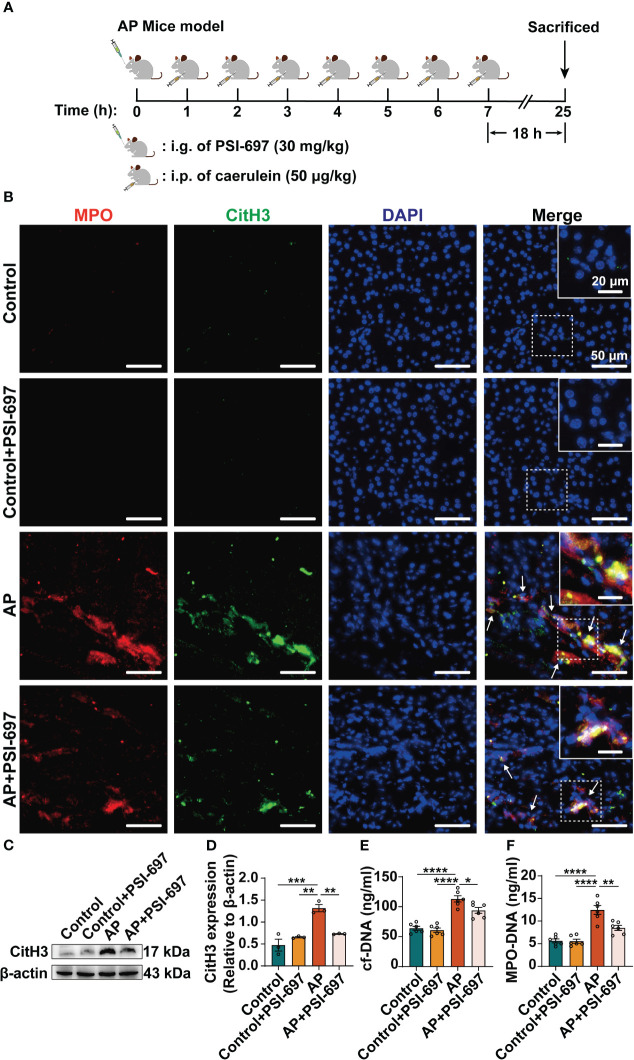
Inhibition of P-selectin binding to PSGL-1 reduces the level of NETs in pancreas of AP mice. **(A)** Drug administration and construction of AP mice. **(B)** Colocalization of MPO (red) with CitH3 (green)in the pancreas of AP mice. The inset box from each group is magnified. Scale bar: 50 μm and 20 μm, respectively. The arrows indicate NETs in pancreas. **(C)** The pancreatic tissue was harvested for lysis and lysates were immunoblotted for CitH3 and β-actin. **(D)** The relative expression levels of CitH3 were quantified. (n=3, **P < 0.01, ***P < 0.001) The serum levels of cf-DNA **(E)** and MPO-DNA complexes **(F)** were determined in mice of different groups. (n=6, *P < 0.05, **P < 0.01, ****P < 0.0001).

We established a caerulein-induced AP mouse model for further study *in vivo* (**Figure 5A**). AP mice were pretreated by PSI-697, an oral inhibitor that inhibit the binding of P-selectin to PSGL-1. In order to assess NETs formation in the pancreas, we evaluated MPO/CitH3 colocalization in tissue sections from the pancreas (**Figure 5B**) and CitH3 expression in pancreatic tissues lysates (**Figures 5C, D**) and found that NETs formation was increased in AP mice and administration of PSI-697 decreased the level of NETs in the pancreas. Moreover, the serum levels of circulating cf-DNA and MPO-DNA complexes in mice were assessed as more objective biomarkers of *in vivo* NETs formation. Serum cf-DNA and MPO-DNA complexes were both elevated in AP mice compared to mice in control group and was decreased in AP mice pretreated with PSI-697 (**Figures 5E, F**). These results indicated that high level of P-selectin induces NETs formation through its ligand PSGL-1*in vivo*. After finding that high level of P-selectin induces NETs formation through PSGL-1, the animals were used to confirm whether PAD4 activity or expression is affected by P-selectin in AP. As shown in **Supplementary Figure 3A**, in PSI-697-pretreated AP mice, decreased PAD4 expression was found in neutrophils infiltrated in pancreas compared with that in the AP group. Compared with AP mice, the percentage of PAD4-positive neutrophils in pancreatic tissues of PSI-697 pretreated AP mice was significantly reduced (**Supplementary Figures 3B, C**). Similarly, the mRNA level of PAD4 in pancreas was lower in PSI-697-pretreated AP mice compared with AP mice (**Supplementary Figure 3D**). Collectively, these data suggest that PAD4 participates in the formation of NETs induced by P-selectin in AP, and its activity and expression are regulated by P-selectin.

### 3.5 Inhibition of P-selectin binding to PSGL-1 alleviates pancreatic histopathological injury and reduces serum enzyme levels in AP

**Figure 6:**
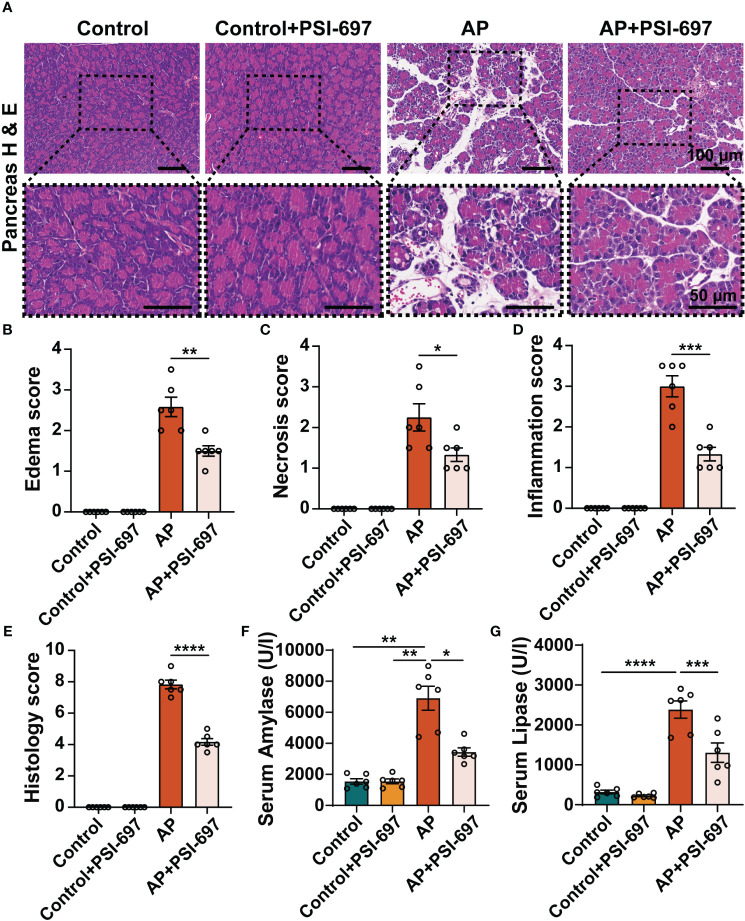
Inhibition of P-selectin binding to PSGL-1 by PSI-697 alleviated pancreatic histopathological injury and reduced serum enzyme levels in AP. **(A)** Representative H&E staining of pancreatic tissues. The inset box from each group is magnified. Scale bar: 100 μm and 50μm, respectively. **(B–E)** Histopathological scores of pancreatic tissues. (n=6, *P < 0.05, **P < 0.01, ***P < 0.001, ****P < 0.000) **(F, G)** Serum amylase and lipase levels in mice. (n=6, *P < 0.05, **P < 0.01, ***P < 0.001, ****P < 0.0001).

Interestingly, we also found that PSI-697 had a protective effect on AP. As shown in **Figure 6**, intraperitoneal injection of caerulein to mice induced pancreas injury, evidenced which was demonstrated by significant elevations of serum amylase and lipase and macroscopic pancreas damage. Pretreatment with PSI-697 significantly ameliorated histological damage of the pancreatic tissue as evidenced by reduced edema, acinar cell necrosis and inflammatory cell infiltration (**Figures 6A–E**). The elevations of serum amylase and lipase induced by intraperitoneal injection of caerulein were also blunted in AP mice pretreated with PSI-697 (**Figures 6F, G**). The above results suggested that PSI-697 might be an effective treatment for AP.

## 4 Discussion

AP is an acute abdominal disease in which an intense systemic inflammatory response plays a crucial role in the development of the disease (24). Clarifying the endogenous mechanisms underlying AP progression could help to clinically treatment and improve prognosis. Besides the classical role of neutrophils, numerous findings suggest that neutrophil-derived NETs play an important role in the pathophysiology of AP (8, 25–27). Based on the role of P-selectin in neutrophil functions, our study focused on the relationship between the highly expressed P-selectin and NETs in AP and found that in human neutrophils P-selectin is an important stimulus of NETs formation, which is consistent with the findings of Etulain et al. in mouse neutrophils (20). Consequently, how the highly expressed P-selectin mediates the intracellular signal and induces the formation of NETs to participate in AP progress is the focus of our research. It has been proved in mice neutrophils that both conjugated and soluble P-selectin could promote NETs formation (20), therefore there is no deliberate distinction between the two types of P-selectin in our study. The P-selectin recombinant protein we used *in vitro* is more similar to soluble P-selectin, while *in vivo*, both two forms of P-selectin coexist.

Many substances stimulate NETs formation, and the diversity of stimuli of NETs also result in heterogeneity in their key pathways (28–34). PAD4 is known to citrullinate histones to mediate chromatin decondensation and NETs formation (35). However, the requirement of PAD4 for NETosis may vary depending on the NETosis stimulus. Studies have highlighted that PAD4 is required for NETosis during both sterile inflammation (36) and bacterial infection (37). We hypothesized that P-selectin has mainly two effects after AP, one affects neutrophil infiltration, and the other stimulates PAD4 activation and expression and leads to increased NETs. In pancreas of AP mice we first observed the increased Ly6G-positive cells, this might be the increasing P-selectin in pancreatic tissue accelerated the infiltration of neutrophils. At the same time, P-selectin reacted on PSGL-1 led to increased expression of PAD4 and promoted the NETs formation. PAD4 is a calcium-specific enzyme containing five calcium-binding sites and the binding of Ca^2+^ to PAD4 would cause a conformational change, leading to the formation of an active site cleft (38). However, intracellular calcium concentrations in activated neutrophils were reported to be well below the levels required for PAD4 activation, suggesting that a cellular mechanism for intracellular calcium regulation within neutrophils is also required to be involved (39, 40). Moreover, Intracellular calcium ions may be the key factor to govern the folding of PAD4, assisting in stabilizing the intermediate state and ensuring the correct and active protein structure (41). It is reported that P-selectin and PSGL-1 induces the intracellular calcium influx of neutrophils (42). PSGL-1 can induce downstream signaling changes, allowing Ca^2+^ flux out of the endoplasmic reticulum into the cytoplasm for exerting various functions (43, 44). Syk, as a key component of multiple intracellular signaling pathways, plays an important role in the signaling pathways involved in NETs formation from a variety of stimuli (45–47), and is also involved in PSGL-1-mediated calcium influx (42). Thus, in this study we speculated and confirmed that it is this process that provides the high calcium environment for PAD4 activation, which ultimately leads to NETs formation. We also excluded the role of ROS in P-selectin-induced NETs in human neutrophils. In the future, we will use neutralizing antibodies as more specific inhibitors and neutrophils from knockout mice for further research.

Since it is difficult to obtain pancreatic tissue samples from human AP cases, we established AP mouse models for further study. In previous studies, we have successfully established the models induced by intraperitoneal injection of caerulein, intraperitoneal injection of caerulein combined with LPS and retrograde pancreatic duct infusion of sodium taurocholate, and their severity increased in turn. However, according to our observation and evaluation, the model induced by caerulein has the highest degree of neutrophil infiltration in pancreatic tissue among the three models. Therefore, we chose the caerulein-induced model to study NETs in AP. Besides, in this study we selected an oral P-selectin inhibitor PSI-697, which can inhibit the binding of P-selectin to PSGL-1 (48), to re-validate this signaling pathway *in vivo*. PSI-697 has been identified to reduce thrombosis in human and animal models in previous studies but the effects on AP have not been studied (49–51). Interestingly, while studying the signal pathway, we found that PSI-697 is an effective treatment for AP. We observed that PSI-697 not only decreases NETs formation in the inflamed pancreas but also had a protective effect against tissue damage in AP, as evidenced by the ability to attenuate the pathological manifestations, amylase and lipase levels in AP. Our results revealed that PSI-697 might be a promising therapeutic strategy for AP, especially in the situation that a Phase 1 clinical trial of PSI-697 has been conducted in healthy individuals and patients with scleritis.

**Figure 7:**
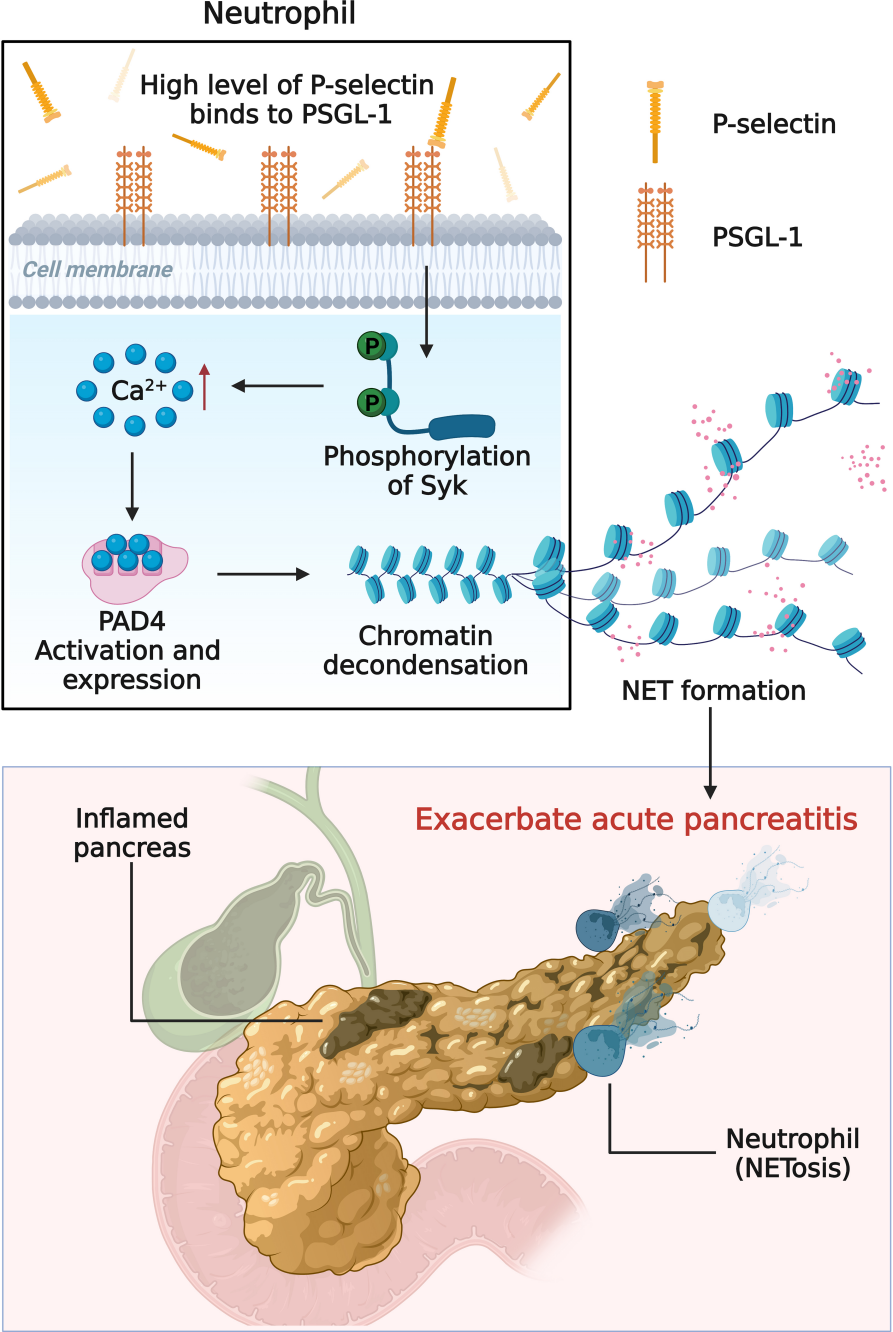
Schematic diagram (created with BioRender.com) depicting the mechanism of high level of P-selectin-induced NETs formation in AP. Upon stimulation of P-selectin, PSGL-1 and its downstream Syk/Ca^2+^/PAD4 signaling pathway induce neutrophils to form NETs and aggravate the deterioration of AP.

In summary, this study showed that P-selectin plays a role in AP progression by inducing NETs formation through PSGL-1/Syk/Ca^2+^/PAD4 signaling pathway, providing a better understanding of the molecular mechanisms of AP (**Figure 7**). In addition, this study preliminarily explored that targeting this pathway might be a promising therapeutic strategy for AP, and other targets in this pathway, such as Syk, will be studied in the future.

## Data availability statement

The original contributions presented in the study are included in the article/**Supplementary Material**. Further inquiries can be directed to the corresponding authors.

## Ethics statement

The studies involving humans were approved by the ethics committee of the Seventh Affiliated Hospital of Sun Yat-Sen University. The participants provided their written informed consent to participate in this study. The animal study was approved by the Ethics Committee of the Shenzhen TopBiotech Co. Ltd. The study was conducted in accordance with the local legislation and institutional requirements.

## Author contributions

QX, MS: performed the experiments, analysis and interpretation of data and manuscript writing. LD, YX, XL, XZ: material or technique support. LL: Supervision, provided clinical guidance and resources. DD: conception, design, supervision, and full responsibility of the study. All authors critically reviewed the article and approved the final manuscript.
