# Supplementary material for: High expression of P-selectin induces neutrophil extracellular traps via the PSGL-1/Syk/Ca2+/PAD4 pathway to exacerbate acute pancreatitis
Source: Front Immunol. 2023 Sep 28;14:1265344. doi: 10.3389/fimmu.2023.1265344 (PMC10568494; doi:10.3389/fimmu.2023.1265344)
Supplement: Supplementary file 1 [file DataSheet_1.pdf]

Supplementary Material

High expression of P-selectin induces neutrophil extracellular traps via the PSGL-1/Syk/Ca^{2+}/PAD4 pathway to exacerbate acute pancreatitis

Qi Xu[1 †], Ming Shi[1 †], Lu Ding[1], Yu Xia[1], Liang Luo[2 *], Xiaofang Lu[3], Xiaoying Zhang[4], David Y.B. Deng[1, 2 *]

[1]Department of Scientific Research Center, Seventh Affiliated Hospital, Sun Yat-Sen University, Shenzhen, China

[2]Department of Critical Care Medicine, Seventh Affiliated Hospital, Sun Yat-Sen University, Shenzhen, China

[3]Department of Pathology, Seventh Affiliated Hospital, Sun Yat-Sen University, Shenzhen, China

[4]Department of Health Management Center, Seventh Affiliated Hospital, Sun Yat-Sen University, Shenzhen, China

*** Correspondence:**

Liang Luo, luoliang@sysush.com

David Y.B. Deng, dengyub@mail.sysu.edu.cn

Supplementary Table 1. Histopathologic scoring criteria of acute pancreatitis in mice(1)

Score	Edema	Necrosis	Inflammation
0	Absent	Absent	0-1 intralobular or perivascular leukocytes/HPF
0.5	Focal occurrence of interlobar septae expansion	Focal occurrence of 1-4 necrotic cells/HPF	2-5 intralobular or perivascular leukocytes/HPF
1	Diffuse occurrence of interlobar septae expansion	Diffuse occurrence of 1-4 necrotic cells/HPF	6-10 intralobular or perivascular leukocytes/HPF
1.5	Focal occurrence of interlobular septae expansion	Focal occurrence of 5-10 necrotic cells/HPF	11-15 intralobular or perivascular leukocytes/HPF
2	Diffuse occurrence of interlobular septae expansion	Diffuse occurrence of 5-10 necrotic cells/HPF	16-20 intralobular or perivascular leukocytes/HPF
2.5	Focal occurrence of interacinar septae expansion	Focal occurrence of 11-16 necrotic cells/HPF	21-25 intralobular or perivascular leukocytes/HPF
3	Diffuse occurrence of interacinar septae expansion	Diffuse occurrence of 11-16 necrotic cells/HPF (foci of confluent necrosis)	26-30 intralobular or perivascular leukocytes/HPF
3.5	Focal occurrence of intercellular septae expansion	focal occurrence of ＞16 necrotic cells/HPF	＞30 leukocytes/HPF or focal microabscesses
4	Diffuse occurrence of intercellular septae expansion	＞16 necrotic cells/HPF (Extensive confluent necrosis)	＞35 leukocytes/HPF or confluent microabscesses

Supplementary Figures

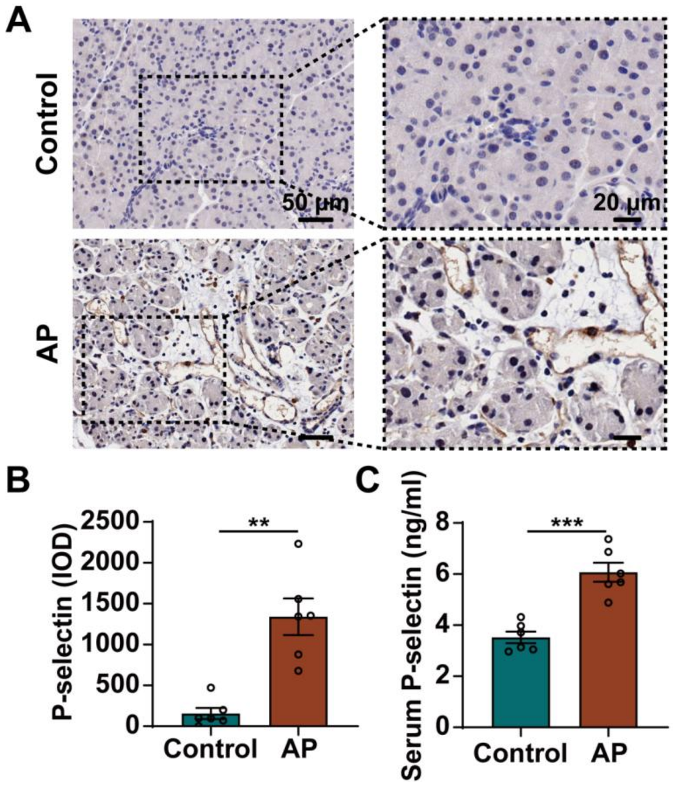

Supplementary Figure 1. Hyperexpression of P-selectin in AP mice. (A) Representative examples of P-selectin IHC staining of pancreas from the control mice and AP mice. The inset box from each group is magnified. Scale bar: 50 μm and 20 μm, respectively. (B) Quantification of IOD of P-selectin immunostaining. (n=6, ***P* < 0.01) (C) Serum P-selectin level in mice of control group and AP group determined by ELISA. (n=6, ****P* < 0.001)

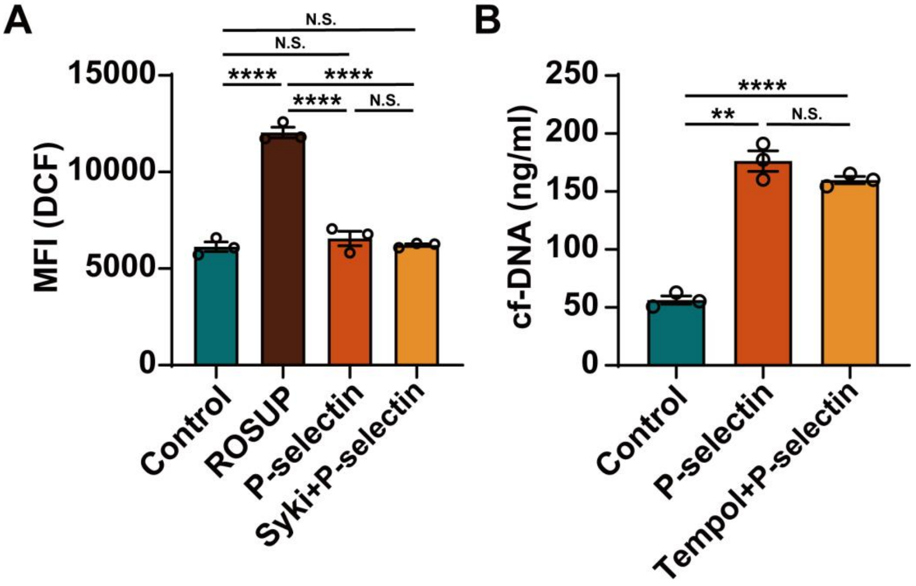

Supplementary Figure 2. Role of ROS production in P-selectin induced NETs formation in human neutrophils. (A) Human neutrophils were treated with P-selectin with Syk inhibitor (Syki, 4 μM of PRT-060318) in the presence of the ROS indicator DCFH-DA (1:5000). Neutrophils treated with ROSUP (1:1000) were used as positive control. The mean fluorescence intensity (MFI) was analyzed by flow cytometry. (n=3, ****P < 0.0001). (B) The level of cf-DNA in Control, P-selectin and Tempol (1 mM) + P-selectin group. The cf-DNA in the supernatants was determined as an indicator of NETs. (n=3, **P < 0.01, ****P < 0.0001)

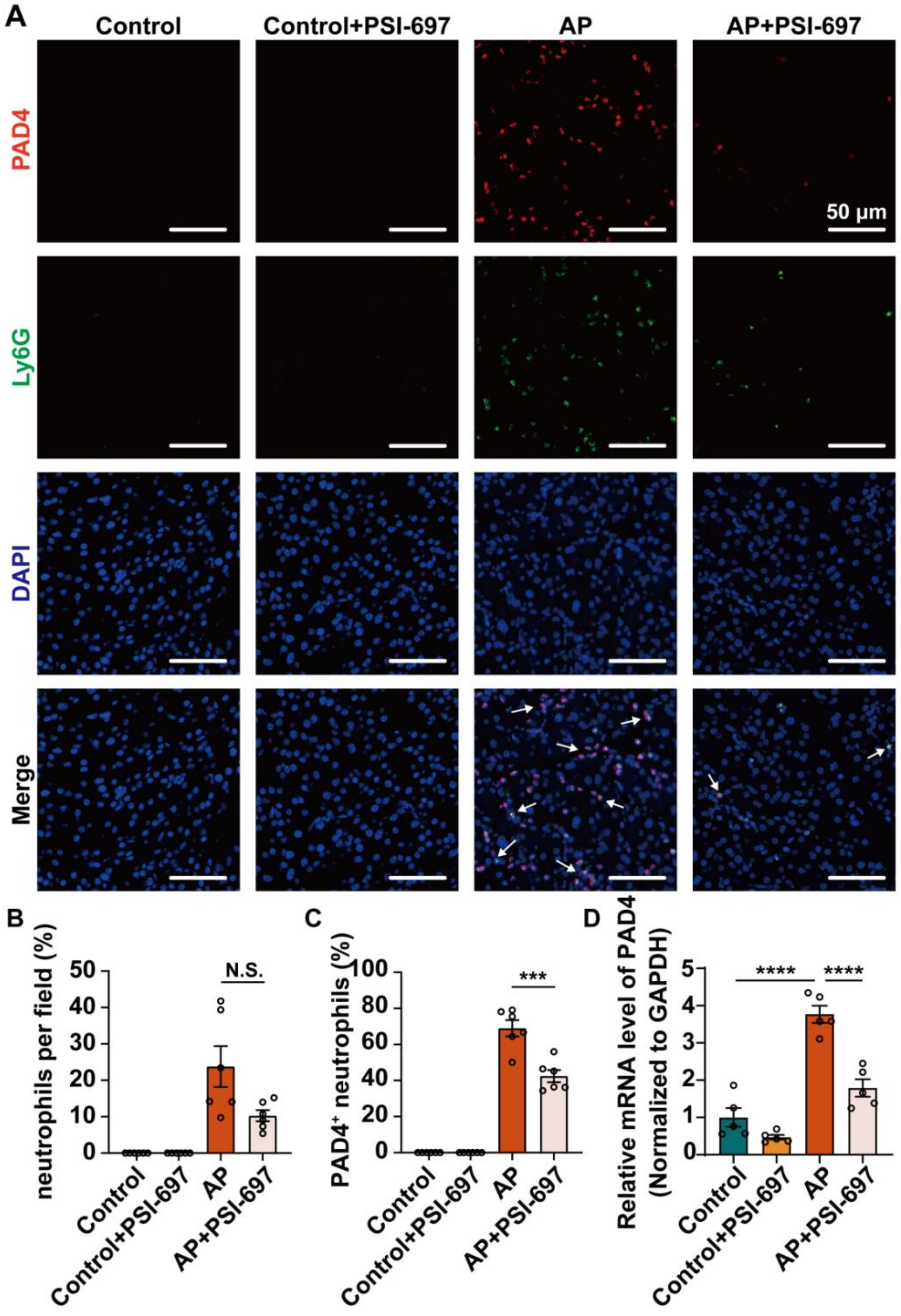

Supplementary Figure 3. Inhibition of P-selectin binding to PSGL-1 by PSI-697 down-regulated the expression of PAD4 in neutrophils infiltrated in pancreatic tissue of AP mice. (A) Individual channel and merged images for PAD4 (red), Ly6G (green) and DAPI (blue) in pancreatic tissue of mice. Ly6G was selected as a marker of mouse neutrophils. The arrows indicate PAD4-positive neutrophils. Scale bar: 50 μm. (B) Quantitative summary of Ly6G^{+} neutrophils per filed of AP and AP+PSI-697 groups. (n=6, N.S., no significance) (C) Quantitative summary of PAD4^{+}

neutrophils of AP and AP+PSI-697 groups. (n=6, ***$P < 0.001$) (D) Quantitative analysis of PAD4 mRNA levels in pancreatic tissues. (n=5, ****$P < 0.0001$)

References

1. Schmidt J, Rattner DW, Lewandrowski K, Compton CC, Mandavilli U, Knoefel WT, et al. A better model of acute pancreatitis for evaluating therapy. Ann Surg (1992) 215: 44-56.
